# Arterial Stiffness Determinants for Primary Cardiovascular Prevention among Healthy Participants

**DOI:** 10.3390/jcm11092512

**Published:** 2022-04-29

**Authors:** Alexandre Vallée

**Affiliations:** Department of Epidemiology-Data-Biostatistics, Delegation of Clinical Research and Innovation (DRCI), Foch Hospital, 92150 Suresnes, France; alexandre.g.vallee@gmail.com

**Keywords:** arterial stiffness index, arterial stiffness, phosphate, albumin, triglycerides, phosphate, albumin, tobacco, BMI, age, mean blood pressure, heart rate, cystatin c, alkaline phosphatase

## Abstract

Background: Arterial stiffness (AS), measured by arterial stiffness index (ASI), can be considered as a major denominator in cardiovascular (CV) diseases. Thus, it remains essential to highlight the risk factors influencing its increase among healthy participants. Methods: According to European consensus, AS is defined as ASI > 10 m/s. The purpose of this study was to investigate the determinants of the arterial stiffness (ASI > 10 m/s) among UK Biobank normotensive and healthy participants without comorbidities and previous CV diseases. Thus, a cross-sectional study was conducted on 22,452 healthy participants. Results: Participants were divided into two groups, i.e., ASI > 10 m/s (*n* = 5782, 25.8%) and ASI < 10 m/s (*n* = 16,670, 74.2%). All the significant univariate covariables were included in the multivariate analysis. The remaining independent factors associated with AS were age (OR = 1.063, threshold = 53.0 years, *p* < 0.001), BMI (OR = 1.0450, threshold = 24.9 kg/m^2^, *p* < 0.001), cystatin c (OR = 1.384, threshold = 0.85 mg/L, *p* = 0.011), phosphate (OR = 2.225, threshold = 1.21 mmol/L, *p* < 0.001), triglycerides (OR = 1.281, threshold = 1.09 mmol/L, *p* < 0.001), mean BP (OR = 1.028, threshold = 91.2 mmHg, *p* < 0.001), HR (OR = 1.007, threshold = 55 bpm, *p* < 0.001), Alkaline phosphate (OR = 1.002, threshold = 67.9 U/L, *p* = 0.004), albumin (OR = 0.973, threshold = 46.0 g/L, *p* < 0.001), gender (male, OR = 1.657, *p* < 0.001) and tobacco use (current, OR = 1.871, *p* < 0.001). Conclusion: AS is associated with multiple parameters which should be investigated in future prospective studies. Determining the markers of increased ASI among healthy participants participates in the management of future CV risk for preventive strategies.

## 1. Introduction

Arterial stiffness (AS) is as a major denominator in target organ damage [1,2]. Numerous noninvasive arterial parameters have been shown to be biomarkers of arterial stiffness [3]. Arterial stiffness is the arteries capacity to expand and contract during the different phases of the cardiac flow. Arterial stiffness can be an integrator of long-lasting arterial wall damage leading to luminal dilation due to an increase in collagen deposition [4]. Arterial stiffness is associated with coronary atherosclerosis [5], cardiovascular (CV) events [6] or inflammatory disorders [7]. Several studies have shown that carotid–femoral (aortic) pulse wave velocity (PWV) can be considered to be the criterion standard for assessment of arterial stiffness. PWV levels are strongly correlated with risk factors such as atherosclerosis [8], hypertension and diabetes [9] and CV diseases [10]. Nevertheless, carotid–femoral PWV measurement is time-consuming and operator dependent.

The arterial stiffness index (ASI) is simple, operator independent, convenient and can be measured by finger photoplethysmography through the utilisation of infrared light (photoplethysmography) to record the volume waveform of the blood into the finger. The shape of the waveform is directly associated to the time it takes for the pulse wave to cross by the arterial tree. These tools could be of interest for rapid estimation of CV risk [11,12]. The European consortium have reported normal references and they stated that 10 m/s was the cutoff for pathological values [9]. However, it remains essential to better understand the factors influencing the increase of ASI, especially in a population without CV comorbidities and CV diseases. Thus, in the context of new challenges in personalised, predictive and preventive medicine, it is essential to understand the harmful factors which could influence CV markers, such as ASI, in healthy participants. Correcting the potential risks of increased ASI should use their precise targeting. Determining the differences between the biological factors of participants with or without ASI > 10 m/s is essential to better understand the underlying pathophysiological mechanisms and, thus, to be able to better manage the patients before the CV event occurs. In this study, the aim was to investigate the biological determinants of ASI > 10 m/s among healthy participants.

## 2. Materials and Methods

### 2.1. UK Biobank Population

The UK Biobank is a prospective cohort for the investigation, prevention, diagnosis and treatment of chronic diseases, such as CV diseases in adults. A total of 502,478 Britons across 22 UK cities from the UK National Health Service Register were included between 2006 and 2010. The cohort was phenotyped and genotyped, by participants who responded to a questionnaire, a computer-assisted interview, including physical and functional measures and blood, urine and saliva samples [13]. Data included socio-economic status, behaviour and lifestyle, a mental health battery, clinical diagnoses and therapies, genetics, imaging and physiological biomarkers from blood and urine samples. The cohort protocol can be found in the literature [14]. All participants provided electronic informed consent and UK Biobank received ethical approval from the North-West Multi-center Research Ethics Committee (MREC) covering the whole of the UK. The study was conducted according to the guidelines of the Declaration of Helsinki, and approved by the North-West–Haydock Research Ethics Committee (protocol code: 21/NW/0157, date of approval: 21 June 2021). For details, visit https://www.ukbiobank.ac.uk/learn-more-about-uk-biobank/about-us/ethics, accessed on 1 January 2022.

### 2.2. Blood Pressure Measurement

Systolic and diastolic blood pressure (SBP, DBP) were measured twice at the assessment centre by the use of an automated BP device (Omron 705 IT electronic blood pressure monitor; OMRON Healthcare Europe B.V. Kruisweg 577 2132 NA Hoofddorp), or manually by the use of a sphygmomanometer with an inflatable cuff in association with a stethoscope if the blood pressure device failed to measure the BP or if the largest inflatable cuff of the device did not fit around the individual’s arm [15].

The participant was sitting in a chair while the measurements were taken. The measurements were carried out by nurses trained in performing BP measurements [16]. Multiple available measurements for one participant were averaged. The Omron 705 IT BP monitor has satisfied the Association for the Advancement of Medical Instrumentation SP10 standard and was validated by the British Hypertension Society protocol, with an overall “A” grade for both SBP and DBP [17]. Nevertheless, automated devices measure higher BP in comparison to manual sphygmomanometers; thus, we adjusted both SBP and DBP, which were measured using the automated device using algorithms [18]:

For SBP, we performed the following algorithm:

$$SBP=3.3171+0.92019\times SBP\;\left(\mathrm{mmHg}\right)+6.02468\times sex\;\left(male=1;\;female=0\right)$$

For DBP, we performed the following algorithm:

$$DBP=14.5647+0.80929\times DBP\;\left(\mathrm{mmHg}\right)+2.01089\times sex\;\left(male=1;\;female=0\right)$$

These adjusted BP values were used for all calculations, including mean BP calculation.

Mean BP was calculated as:

$$mean\;BP=\frac{\left(SBP+2\times DBP\right)}{3}$$

### 2.3. Arterial Stiffness Measurement

Pulse wave arterial stiffness index (ASI) was measured by a non-invasive method during a volunteer’s visit to a UK Biobank Assessment Centre. Pulse waveform was taken by clipping a photoplethysmograph transducer (PulseTrace PCA 2^TM^, CareFusion, San Diego, CA, USA) to the rested volunteer’s finger (any finger or thumb, mainly the index finger). Volunteers were asked to breathe in and out slowly five times in a relaxed fashion and readings were taken over a 10–15 s period. ASI is performed from a single peripheral pulse waveform. The carotid-to-femoral pulse transit time was estimated from the dicrotic waveform as the time difference between a forward compound when the pressure is transmitted from the left ventricle to the finger and a reflected or backward compound as the wave is transmitted from the heart to lower body via the aorta [19]. ASI was estimated in meters per second (m/s) as H/PTT. H is the individual’s height, and PTT is the pulse transit time or the peak-to-peak time between the systolic and diastolic wave peaks in the dicrotic waveform [19]. This methodology has been validated by comparing it with carotid–femoral PWV. These studies concluded that both measurement methods were highly correlated. ASI was a simple, operator-independent, non-expensive and rapid method [11,20,21]. We excluded extreme outlier ASI values from the analyses (defined as mean +/− 5 * standard deviation).

### 2.4. Laboratory and Clinical Parameters

Hypertension was defined as systolic blood pressure (SBP) of at least 140 mmHg and/or diastolic BP (DBP) of at least 90 mmHg, according to guidelines by the European Society of Cardiology, and/or antihypertensive drug use [20] or hypertension diagnosed by a doctor (reported by in questionnaire, as “has a doctor ever told you that you have had any of the following conditions (i.e., high blood pressure)?”. Diabetes status was defined as either receipt of anti-diabetic medication or diabetes diagnosed by a doctor (reported by in questionnaire, as “has a doctor ever told you that you have diabetes?”) or a fasting glucose concentration ≥ 7 mmol/L. Dyslipidemia was defined as having a fasting plasma total-cholesterol or triglycerides level of ≥6.61 mmol/L (255 mg/dL) or >1.7 mmol/L (150 mg/dL), respectively, or having statins medication. Calculated glomerular filtration rate (GFR) (by MDRD formula, MDRD: modification of diet in renal disease, by mL/min/1.73 m^2^; GFR < 60 mL/min/1.73 m^2^ defined chronic kidney disease (CKD)). Current tobacco smokers were defined as participants who responded “yes, on most or all days” at the question “do you smoke tobacco now”. CV diseases were defined by heart attack, angina and stroke, as diagnosis by a doctor and reported in questionnaires (by the question, “has a doctor ever told you that you have had any of the following conditions?”). Obesity was defined as a body mass index (BMI) higher than 30 kg/m^2^.

### 2.5. Study Population

**Figure 1:**
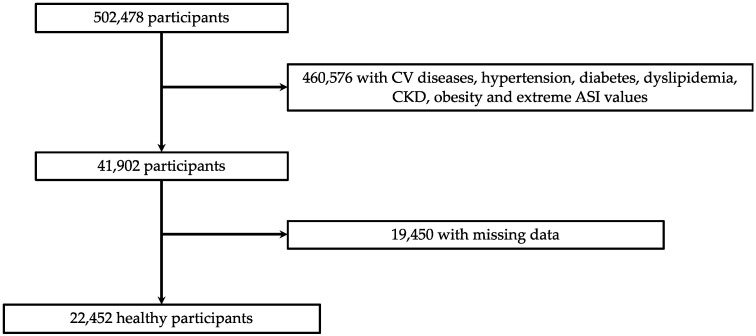
Flowchart. CV: cardiovascular; CKD: chronic kidney disease; ASI: arterial stiffness index.

Of the 502,478 participants, 460,576 were excluded due to CV diseases, hypertension, diabetes, dyslipidemia, CKD, obesity or extreme values of ASI. We excluded extreme outlier ASI values from the analyses (defined as mean +/− 5 * standard deviation). Then 19,450 participants were excluded for data missing and finally 22,452 healthy participants were included in the study (Figure 1).

### 2.6. Statistical Analysis

Characteristics of the study population were described as the means with standard deviation (SD) for continuous variables. Comparisons between groups were performed using Student’s test for continuous variables. Pearson’s Chi-2 test was performed for categorical variables. An ASI superior to 10 m/s was defined as arterial stiffness according to the European consortium [9]. Firstly, univariate associations were performed between ASI > 10 m/s and various clinical parameters and biomarkers. Secondly, only the significant univariate covariates were included in the multivariate model. A forward–backward multiple logistic regression model was performed to discriminate independent factors (*p* < 0.05) associated with arterial stiffness. The accuracy and the receiver operating characteristics (ROC) curve were measured [21]. An ROC graph is a method for visualising and selecting classifiers based on their performance [22]. The area under the curve (AUC) of the classifier can be described as the probability of the classifier to rank a randomly selected positive result the highest predictive accuracy [23].

For each independent classifier of the logistic multivariate analysis, the ability of the logistic regression models to allow discrimination was quantified by the area under the ROC curve (AUC).

The maximum Youden index, performed as:

$$J=max_{c}\left[S_{e}\left(c\right)+S_{p}\left(c\right)-1\right]$$

was chosen to determine the optimal decision thresholds (*c*) for the discrimination.

Statistics were performed using SAS software (version 9.4; SAS Institute, Carry, NC, USA). A *p* value < 0.05 was considered statistically significant.

## 3. Results

The characteristics of the 22,452 healthy participants were shown in Table 1. Participants were divided into two groups, i.e., ASI > 10 m/s (*n* = 5782, 25.8%) and ASI < 10 m/s (*n* = 16,670, 74.2%).

The two groups were significantly different for all the covariates, except for calcium (*p* = 0.580), lipoprotein (a) (*p* = 0.924) and total bilirubin (*p* = 0.295). The group with ASI > 10 m/s presented an average of 12.0 m/s whereas the group with ASI < 10 m/s had an average equal to 7.1 m/s (*p* < 0.001). Participants with ASI > 10 m/s were older (55.9 years vs. 51.3 years, *p* < 0.001), displayed higher tobacco use (9.9% vs. 5.7%, *p* < 0.001) and there were fewer women (51.9% vs. 68.4%, *p* < 0.001).

All the significant univariate covariables were included in the multivariate analysis. The remaining independent factors were age (OR = 1.063, *p* < 0.001), BMI (OR = 1.0450, *p* < 0.001), cystatin c (OR = 1.384, *p* = 0.011), phosphate (OR = 2.225, *p* < 0.001), triglycerides (OR = 1.281, *p* < 0.001), mean BP (OR = 1.028, *p* < 0.001), HR (OR = 1.007, *p* < 0.001), alkaline phosphate (OR = 1.002, *p* = 0.004), albumin (OR = 0.973, *p* < 0.001), gender (men, OR = 1.657, *p* < 0.001) and tobacco status (current, OR = 1.871, *p* < 0.001) (Table 2).

**Figure 2:**
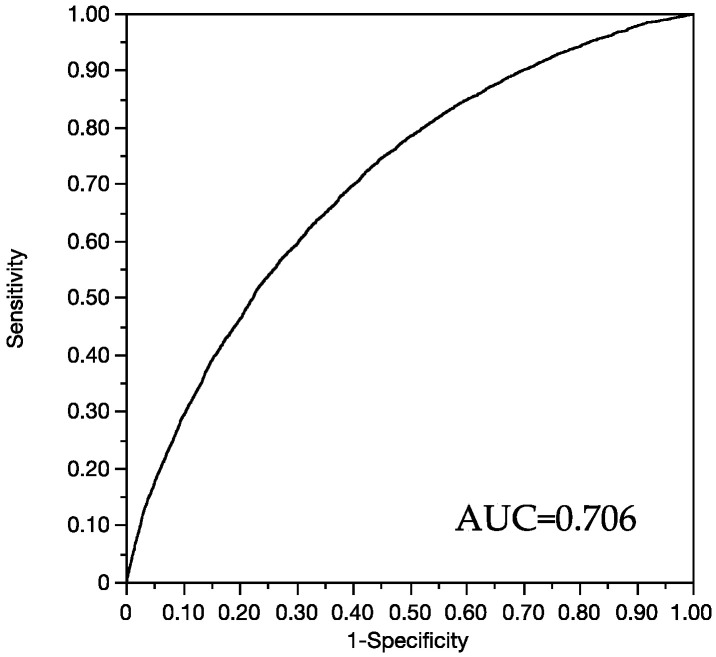
AUC (area under the ROC curve) of the multivariate analysis (cf. Table 2).

The accuracy (AUC) of the multivariate model was 0.706 (Figure 2).

For each independent parameter, Youden indexes were calculated to performed threshold values to discriminate ASI > 10 m/s or not (Table 3). Cutoff values for determining arterial stiffness corresponded to age superior to 53.0 years (AUC = 0.663, *p* < 0.001), BMI superior to 24.9 kg/m^2^ (AUC = 0.567, *p* < 0.001), cystatin c superior to 0.85 mg/L (AUC = 0.610, *p* < 0.001), phosphate superior to 1.21 mmol/L (AUC = 0.516, *p* < 0.001), triglycerides superior to 1.09 mmol/L (AUC = 0.574, *p* < 0.001), mean BP superior to 91.2 mmHg (AUC = 0.606, *p* < 0.001), heart rate superior to 55 bpm (AUC = 0.514, *p* < 0.001), Alkaline phosphatase superior to 67.9 U/L (AUC = 0.577, *p* < 0.001), but albumin inferior to 46.0 g/L (AUC = 0.540, *p* < 0.001), male gender (AUC = 0.583, *p* < 0.001) and current smoking status (AUC = 0.502, *p* < 0.001).

## 4. Discussion

This study showed that arterial stiffness was present in 25.8% of the normotensive and healthy population. This result appears to be concordant with previous studies showing similar rates [24,25]. Moreover, the multivariate analysis showed that four well-known independent risk factors were predictive of arterial stiffness in normotensive and healthy subjects; these were age, gender, mean blood pressure and heart rate [26].

Arterial stiffness, along with blood pressure, increases with age in both genders and with increase in mean BP [27] contributing to the promotion of vascular thickening and fibrosis [28]. The main mechanism involved is alterations in the structure of the extracellular matrix (ECM), with enhancement of collagen deposition and the increase in elastin breakdown [29]. Recent findings have shown the implication of the vascular smooth muscle cell (VSMC) as a direct source of arterial stiffness through the alteration in the cytoskeleton and integrin interactions with the ECM [30]. Moreover, vascular oxidative stress can derived from mitochondrial dysregulation and increased superoxide production as processes which can enhance arterial stiffening with aging [31].

Gender is a well-known pejorative factor with a pejorative pathway for men [28,32]. However, with aging this relationship remains complex with women showing a more rapid increase in stiffening after the onset of the menopause, consistent with the idea that the removal of estrogen can contribute to aging-associated arterial stiffening in females [33].

The increase in sympathetic activity showed by elevated HR can reduce arterial distensibility. The trophic effect of sympathetic nervous system can influence modification in arterial wall tissue and, therefore, the arterial wall structure in a way that favors its less-extensible components and increases its thickness [30]. Nevertheless, the role of HR remains controversial [34] due to HR dependence on PWV decrease at higher levels of BP [35].

This study shows that arterial stiffness presented several biological parameters, including albumin, alkaline phosphatase, phosphate, cystatin c and triglycerides, in association with BMI and tobacco status. By performing thresholds, the results may help to discriminate healthy participants with high risk of arterial stiffness, and this can participate in the implementation of primary prevention focused on biological parameters and on behaviors such as tobacco use. Nevertheless, even if the multiple covariates regression model showed an AUC = 0.706, each determinant presented low performance to determine AS. This could be explained by the healthy aspect of participants where each parameter remained little associated with AS before CV events occurred. However, our modelling performances were consistent with previous works in healthy populations [36,37].

Current tobacco smokers were mainly presented in cluster number 8 but in which 37% of the participants had arterial stiffness. The role of tobacco remains unclear in arterial stiffness in this study. Nevertheless, numerous studies have explained the possible link between tobacco use and arterial stiffness [38]. Active tobacco smoking is associated with increased arterial wall thickness and arterial stiffness [39], suggesting that active tobacco smoking accelerates atherosclerosis, reduces endothelium-dependent arterial dilatation [39] and increases the stiffness of muscular arteries [40].

BMI and arterial stiffness are closely associated [41,42]. Increase in BMI can be a factor for arterial remodeling leading to a modification in haemodynamic and arterial changes detrimental to vascular function [43] and vascular endothelial wall [44].

Some studies have independently associated serum Phosphate with arterial stiffness [45,46,47] in population with or without CKD [45,48]. The biological effect of serum Phosphate on arterial stiffness is complex and presents multiple influences, but presents the highest OR (OR = 2.225) in this study. In the presence of a high level of serum phosphate, vascular smooth muscle cells retain their ability to mineralise [49]. Phosphate in association with calcium levels induce VSMC death and apoptotic body release (with inflammation), as well as matrix vesicle release, leading to calcification [50]. Moreover, increased phosphate levels suppress vitamin D synthesis, leading to an increase in arterial calcification [51]. Several findings have shown that high serum phosphate levels are associated with high all-cause mortality; thus, phosphate could accelerate aging, a major determinant of arterial stiffness [52]. Recent studies have shown that phosphate reduction may improve vascular end-points, especially in CKD patients [53]. Furthermore, medial arterial calcification is characterised by disseminated and progressive precipitation of calcium phosphate within the medial layer, a prolonged and clinically silent course, and compromise of haemodynamics associated with chronic limb-threatening ischaemia. The accumulation of calcium phosphate with the formation of hydroxyapatite crystals results in progressive petrification of the medial layer of the arterial wall [54].

Similarly, triglycerides are well-established as risk factor for arterial stiffness [55,56]. The subendothelial space can be invaded by cholesterol-enriched remnant byproducts following the hydrolysis of exogenously derived chylomicrons or endogenously secreted by very-low-density lipoproteins [57]. Furthermore, elevated triglyceride levels can promote atherosclerosis through the scavenger receptor class B Type 1 (SR-BI) by impairing the capacity of high-density lipoprotein to deliver cholesteryl esters [58]. High levels of triglycerides can induce inflammation and oxidative stress to enhance adhesion molecule expression and foam cell formation, to stimulate the toxicity of smooth muscle [59] and to increase the release of endothelin-1 responsible for the development of atherosclerosis [60].

Cystatin c is a cysteine protease inhibitor which has been an early and sensitive marker of renal function [61]. Cystatin c could be considered as an integrator behavioural factor. Cystatin c is mainly associated with several medical conditions, including metabolic syndrome, diabetes, physical activity, smoking, diet and drinking [62]. Previous studies have shown that cystatin c was associated with arterial stiffness in the general population [63,64].

Alkaline phosphatase is a main factor of hepatobiliary or bone disorders and has been found to be correlated with CV diseases [65]. The relationship between alkaline phosphatase and arterial stiffness remains unclear, but several hypotheses can be made. Alkaline phosphatase catalyzes the hydrolysis of inorganic pyrophosphate, downregulating the expression of hydroxyapatite and the level of inorganic pyrophosphate to promote vascular calcification [66]. Furthermore, alkaline phosphatase is associated with chronic inflammation. During the process of chronic inflammation, tumor necrosis factor (TNF)-*α* and interleukin (IL)-1 *β* levels are increased, thus leading to the stimulation of alkaline phosphatase activity in vascular smooth muscle cells [67].

## 5. Limitations

The main strength of this study is the very large sample size of the cohort. However, the cross-sectional observational design limits the relationship of causality. Reverse causation cannot be ruled out. A potential limitation could stem from the utilisation of the Pulse Trace device to measure arterial stiffness on account of greater variability in ASI values relative to other available devices [68]. The UK Biobank study showed a low response rate of 5.5% and possible volunteer bias may be involved. Nevertheless, given the large sample size and high internal validity, these are unlikely to affect the reported associations [69,70]. Our study presents some limitations, such as in medical history and comorbidities, which have been collected by self-reporting and physician assertion during medical examination in health centers. In addition, the study cohort consisted of middle-aged European participants, so these findings may not be generalisable to other age groups and ethnic populations.

## 6. Conclusions

In the normotensive and healthy participants, we observed that a quarter presented arterial stiffness (i.e., ASI > 10 m/s). The different parameters observed showed that arterial stiffness is associated with multiple parameters. In healthy participants, arterial stiffening is associated with well-known parameters as gender, aging, mean blood pressure, tobacco use, triglycerides, body mass index and heart rate. However, other complex associations should be highlighted, such as cystatin c, phosphate, albumin and alkaline phosphatase. Future clinical trials may involve these parameters to better understand their associations with arterial stiffness and their role in the increase of vascular stiffening in healthy subjects. This phenotyping could optimise clinical trial designs.

## Figures and Tables

**Table 1:** Characteristics of the study population according to arterial stiffness status (ASI > or < to 10 m/s).

	ASI < 10 m/s		ASI > 10 m/s		
	*n* = 16,670		*n* = 5782		*p* Value
Gender (female)	11,396	68.4%	2998	51.9%	<0.001
Tobacco (yes)	943	5.7%	576	9.9%	<0.001
Age (years)	51.3	7.8	55.9	7.7	<0.001
Arterial Stiffness index (ASI), m/s	7.12	1.48	12.08	1.81	<0.001
Systolic Blood Pressure (SBP), mmHg	118.7	11.3	122.6	10.5	<0.001
Diastolic Blood Pressure (DBP), mmHg	76.2	6.1	78.2	5.8	<0.001
Mean Blood Pressure (MBP), mmHg	90.4	7.2	93.1	6.7	<0.001
Heart Rate (HR), bpm	65	10	66	9	<0.001
Body Mass index (BMI), kg/m^2^	24.2	2.6	24.8	2.6	<0.001
Alanine Aminotransferase (ALT), U/L	18.3	9.7	19.7	10.4	<0.001
Albumin, g/L	45.4	2.5	45.1	2.4	<0.001
Alkaline Phosphatase, U/L	74.9	22.7	80.1	23.1	<0.001
Apolipoprotein A1, g/L	1.59	0.25	1.57	0.25	<0.001
Apolipoprotein B, g/L	0.92	0.16	0.96	0.15	<0.001
Aspartate aminotransferase (AST), U/L	24.0	8.2	24.9	10.6	<0.001
Calcium, mmol/L	2.3	0.08	2.37	0.09	0.580
Creatine, micromole/L	68.8	12.8	71.4	13.3	<0.001
C reactive protein (CRP), mg/L	1.60	3.39	1.98	4.14	<0.001
Cystatin c, mg/L	0.82	0.11	0.87	0.12	<0.001
Gamma glutamyl transferase, U/L	24.7	25.0	28.3	29.8	<0.001
Glucose, mmol/L	4.89	0.48	4.93	0.47	<0.001
HDL cholesterol, mmol/L	1.59	0.34	1.53	0.34	<0.001
Total cholesterol, mmol/L	5.32	0.72	5.41	0.71	<0.001
LDL cholesterol, mmol/L	3.23	0.56	3.34	0.55	<0.001
Triglycerides, mmol/L	1.03	0.30	1.11	0.30	<0.001
Lipoprotein (a), nmol/L	43.4	47.8	43.5	47.7	0.924
Phosphate, mmol/L	1.19	0.15	1.20	0.15	<0.001
Testosterone, nmol/L	5.10	6.31	7.14	6.78	<0.001
Total bilirubin, micromol/L	9.68	4.86	9.60	4.61	0.295
Insulin like Growth Factor (IGF), nmol/L	22.8	5.5	21.8	5.44	<0.001
Urate, mmol/L	266.2	65.4	287.1	70.1	<0.001
Vitamin D, nmol/L	52.8	22.3	53.5	21.8	0.045
Glomerular filtration rate (GFR), mL/min/1.73 m^2^	93.3	15.9	94.2	16.0	<0.001

For continuous covariates: mean and standard deviation, or for categorical covariates: *n* and percentage.

**Table 2:** Forward–backward multivariate logistic regression model for arterial stiffness. CI: confidence interval.

Parameters	Odds Ratio	95% CI	*p* Value
Age (years)	1.063	[1.058–1.068]	<0.001
Body Mass index (BMI), kg/m^2^	1.050	[1.037–1.063]	<0.001
Cystatin c, mg/L	1.384	[1.027–1.863]	0.011
Phosphate, mmol/L	2.225	[1.784–2.781]	<0.001
Triglycerides, mmol/L	1.281	[1.150–1.426]	<0.001
Mean Blood Pressure (MBP), mmHg	1.028	[1.023–1.034]	<0.001
Heart Rate (HR), bpm	1.007	[1.004–1.011]	<0.001
Alkaline Phosphatase, U/L	1.002	[1.001–1.003]	0.004
Albumin, g/L	0.973	[0.961–0.986]	<0.001
Gender (male)	1.657	[1.538–1.784]	<0.001
Tobacco (yes)	1.871	[1.663–2.105]	<0.001

**Table 3:** Thresholds values and their performance for each independent parameter to discriminate ASI > 10 m/s.

Parameters	Thresholds	AUC	Sensitivity	Specificity	Accuracy	*p* Value
Age (years)	53.00	0.663	65.8%	59.2%	60.9%	<0.001
Body Mass index (BMI), kg/m^2^	24.91	0.567	50.4%	60.0%	57.6%	<0.001
Cystatin c, mg/L	0.85	0.610	55.4%	61.7%	60.0%	<0.001
Phosphate, mmol/L	1.21	0.516	47.1%	55.7%	53.5%	<0.001
Triglycerides, mmol/L	1.09	0.574	51.1%	59.7%	57.5%	<0.001
Mean Blood Pressure (MBP), mmHg	91.24	0.606	63.1%	53.0%	55.6%	<0.001
Heart Rate (HR), bpm	55.0	0.514	89.9%	11.9%	32.0%	<0.001
Alkaline Phosphatase, U/L	67.9	0.577	70.0%	41.9%	49.2%	<0.001
Albumin, g/L	46.0	0.540	65.1%	41.3%	47.4%	<0.001
Gender (male)	-	0.583	48.2%	68.4%	63.2%	<0.001
Tobacco (yes)	-	0.502	52.4%	64/8%	61.6%	<0.001

## Data Availability

Not applicable.
